# Promoting Dentin Bridge Formation Through N-Acetyl-L-Cysteine Application in Rat Molar Pulpotomy: An Experimental Study

**DOI:** 10.3390/jfb16040117

**Published:** 2025-03-27

**Authors:** Kota Takagi, Koichi Nakamura, Yoshitaka Yoshimura, Yasutaka Yawaka

**Affiliations:** 1 Department of Dentistry for Children and Disabled Persons, Graduate School of Dental Medicine, Hokkaido University, Kita 13 Nishi 7, Kita-ku, Sapporo 060-8586, Japan; mistersmile0804@den.hokudai.ac.jp (K.T.); yawaka@den.hokudai.ac.jp (Y.Y.); 2 Department of Molecular Cell Pharmacology, Graduate School of Dental Medicine, Hokkaido University, Kita 13 Nishi 7, Kita-ku, Sapporo 060-8586, Japan; yoshi@den.hokudai.ac.jp

**Keywords:** mineral trioxide aggregate, N-acetyl-L-cysteine, pulp capping material, pulpotomy

## Abstract

Pulpotomy is performed when tooth decay reaches the dental pulp or when the crown is fractured due to trauma. Mineral trioxide aggregate (MTA) is commonly used in pulpotomy, but its prognosis can be variable. N-acetyl-L-cysteine (NAC), an antioxidant amino acid, has garnered attention due to its potential benefits. This study aimed to investigate the effects of MTA and NAC on pulpotomy outcomes. We used Sprague Dawley rat maxillary molars to perform pulpotomy and employed Superbond C&B, MTA, and MTA mixed with NAC (MTA–NAC) for pulp capping. We obtained tissue sections 3 and 7 days postpulpotomy, conducting histological analysis by examining the morphology of pulp tissue and assessing dentin sialophosphoprotein (DSPP) and osteopontin expression levels. At 3 days postpulpotomy, MTA and MTA–NAC reduced the inflammatory response. At 7 days postpulpotomy, dentin bridge formation was observed following MTA–NAC application, and although MTA resulted in DSPP- and osteopontin-positive areas, these areas were more extensive following MTA–NAC application. Given that adding NAC to MTA enhanced dentin bridge formation, MTA–NAC appears to be a superior option for pulp capping.

## 1. Introduction

Dental caries, a disease preventable through lifestyle modifications and fluoride application [1], persists as a significant health challenge, particularly among children. Early childhood caries (ECC), defined by the presence of at least one decayed, missing, or filled tooth in children under six, has emerged as a pressing social concern [2]. Deciduous teeth, characterized by their thinner enamel and dentin layers, are not only more susceptible to carious lesions, but also prone to rapid progression to pulpal involvement [3]. Consequently, pediatric dental caries treatment frequently necessitates pulpal interventions [4]. When pulpitis is confined to the coronal pulp in deciduous teeth, pulpotomy—the removal of the infected coronal portion while preserving the vital radicular pulp—is the standard of care [5]. The rationale behind preserving the radicular pulp lies in its crucial biological function in maintaining dentin vitality and contributing to long-term tooth survival [6]. Therefore, the induction of a dentin bridge, a reparative hard tissue barrier formed by the pulp’s odontoblast-like cells, is paramount for successful pulpotomy outcomes [7].

In recent years, mineral trioxide aggregate (MTA) has gained widespread acceptance as a superior pulp capping material for pulpotomy procedures. MTA’s biocompatibility and its ability to release calcium and hydroxide ions in a moist environment have been extensively documented [8]. This sustained release results in a high-pH environment, fostering the formation of a superficial necrotic layer on the pulp surface, which subsequently facilitates the recruitment of inflammatory cells and the formation of granulation tissue [9]. The ensuing healing process involves the differentiation of odontoblast-like cells, leading to the deposition of a dentin bridge [10]. While MTA has demonstrated remarkable success in numerous clinical scenarios, its application is not without limitations [11,12]. The high alkalinity of MTA can provoke an intense inflammatory response in the pulp, potentially leading to adverse clinical outcomes, especially in cases of severe inflammation or when precise placement is challenging.

Beyond MTA, a spectrum of pulp capping materials has been explored in clinical practice. Calcium hydroxide, historically favored for its high pH and antimicrobial properties, has been used for many years, but it poses some questions regarding long-term success [13]. Furthermore, advancements in biomaterials have introduced bioceramic materials and growth-factor-based therapies, aiming to enhance pulp regeneration. These novel materials, similar to MTA, offer excellent biocompatibility and promise to promote pulp tissue repair. Nevertheless, challenges related to material handling and long-term stability persist [14].

N-acetyl-L-cysteine (NAC), an antioxidant amino acid and a precursor to glutathione, presents an intriguing alternative [15]. NAC’s acidic properties in aqueous solutions, coupled with its antimicrobial activity [16,17] and anti-inflammatory effects through TNF-α suppression [18], make it a promising candidate for pulp capping applications. By mitigating bacterial infection and modulating inflammatory responses, NAC could potentially enhance pulp healing and dentin bridge formation. However, to date, the application of NAC in pulpotomy procedures remains largely unexplored.

This study aims to investigate the efficacy of NAC when combined with MTA as a novel pulp capping material. Specifically, we performed pulpotomies on rat maxillary first molars, employing MTA and a mixture of MTA with NAC (MTA–NAC) as capping agents. We hypothesized that the addition of NAC would modulate the inflammatory response and promote superior dentin bridge formation compared to MTA alone. Our study involved histological and immunohistochemical evaluations to assess pulp tissue responses, along with an examination of the physicochemical properties of the combined material. The findings from this research are expected to provide valuable insights into developing more effective pulp capping strategies, thereby improving clinical outcomes in pediatric dental care.

## 2. Materials and Methods

### 2.1. pH Measurement

pH measurement of MTA powder dissolved in culture medium (10 mL) was conducted. For the MTA group, MTA powder (0.2 g) was dissolved in the culture medium. For the MTA–NAC group, MTA powder (0.2 g) was mixed with 250 mM NAC solution and then dissolved in the culture medium. Following dissolution, the pH of each group was measured using a pH meter (HORIBA F-2000PI, HORIBA, Ltd., Kyoto, Japan).

### 2.2. Pulpotomy Procedures

Four-week-old male Sprague Dawley rats (Sankyo Lab Service, Inc., Tokyo, Japan) were used in all experiments. A total of 12 animals were used in this study, with 3 animals allocated to each experimental group. The control group consisted of contralateral teeth from the same individual rats. The dental materials employed in this study are presented in Table 1.

The rats were initially anesthetized using isoflurane (Pfizer Inc., Tokyo, Japan) inhalation before administering an intraperitoneal injection of a three-drug mixture: medetomidine hydrochloride (0.15 mg/kg; Dolbene^®^ Kyoritsu Seiyaku, Tokyo, Japan), midazolam (2 mg/kg; Dolmicam^®^ Maruishi Pharmaceutical, Osaka, Japan), and butorphanol (2.5 mg/kg; Betrufal^®^ MeijiSeika Pharma, Tokyo, Japan). Once anesthetized, the rats were secured with a net and placed on a treatment table for the procedure. The bilateral maxillary first molars were disinfected with 70% ethanol, and then, the occlusal surface was drilled with a #1/2 sterile steel round bar to create an area of pulp exposure approximately 1.0 mm in diameter. The pulp was removed using a spoon excavator, and a pulpotomy was performed. Following pulpotomy, the pulp was rinsed with sterile saline solution, and hemostasis was achieved using sterile paper points (#40). After hemostasis was confirmed, the pulp was capped with either MTA cement (Mielle^®^, YAMAKIN, Kochi, Japan) or MTA cement mixed with 50 mg of NAC (MTA and MTA–NAC, respectively; Table 2). Then, the pulp capping material was covered with Superbond C&B (Sun Medical, Shiga, Japan). For the control group, teeth were covered directly with Superbond C&B without a pulp capping material. In these control teeth, the pulp was exposed, and pulp tissue was removed. The intention of having this control group was to evaluate the effect of Superbond C&B without pulp capping material on the pulp tissue.

### 2.3. Tissue Preparation and Histological and Immunohistological Staining

Three days or seven days postpulpotomy, the rats were euthanized using isoflurane. The maxillary bones were then removed and fixed in 4% paraformaldehyde phosphate buffer (Wako Pure Chemical Industries, Osaka, Japan) for 24 h. Subsequently, the tooth specimens were demineralized in 10% ethylenediaminetetraacetic acid solution for 4 weeks, embedded in paraffin using standard procedures, and prepared into 5 µm thick sagittal sections. Subsequently, hematoxylin and eosin (H–E) staining was performed for histological observations under an optical microscope. For immunostaining, 5 µm thick sections were deparaffinized, and endogenous peroxidase activity was blocked with 3% H_2_O_2_ for 5 min. Then, the sections were blocked with 5% bovine serum albumin solution (Sigma-Aldrich, MO, USA) for 10 min at room temperature before incubating them with antidentin sialophosphoprotein (DSPP) antibody (anti-DSPP, rabbit-poly, BS 71212; Funakoshi, Tokyo, Japan) and antiosteopontin (antiosteopontin, rabbit-poly, ab 8448; Abcam, Milton, UK) for 2 h at room temperature. Peroxidase-conjugated antirabbit IgG antibody (histofine polyclonal goat antirabbit IgG, Nichirei Bioscience Corporation, Tokyo, Japan) was used as a secondary antibody for 1 h of incubation at room temperature. Then, the cells were chromogenized with DAB substrate (Takara Bio Inc., Shiga, Japan) for 10 s, and nuclear staining was performed with Meyer hematoxylin. Dehydrated and sealed sections were observed under an optical microscope following standard procedures.

## 3. Results

### 3.1. pH Measurement Results

**Figure 1:**
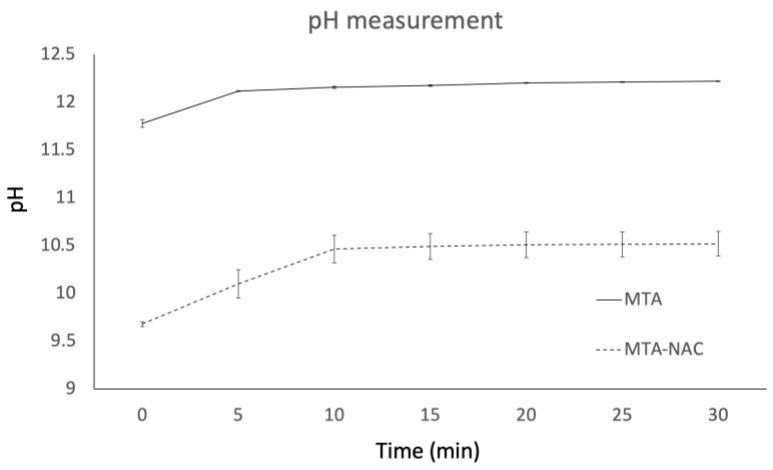
Time-dependent changes in pH of MTA and MTA-NAC solutions.

The pH of MTA cement powder dissolved in culture medium was initially alkaline, measuring 12.22. This high pH value is consistent with the known alkalinity of MTA. However, the addition of 250 mM NAC to MTA cement significantly reduced the pH to 10.60. This reduction in pH suggests that NAC effectively mitigates the strong alkalinity associated with MTA. Furthermore, the time-dependent pH changes in the MTA and MTA-NAC solutions were monitored (Figure 1). The MTA group maintained a high pH throughout the observation period, while the MTA-NAC group exhibited a consistently lower pH, indicating that the pH-lowering effect of NAC was sustained over time.

### 3.2. Histological Examination

#### 3.2.1. Pulp Changes at Three Days Postpulpotomy

**Figure 2:**
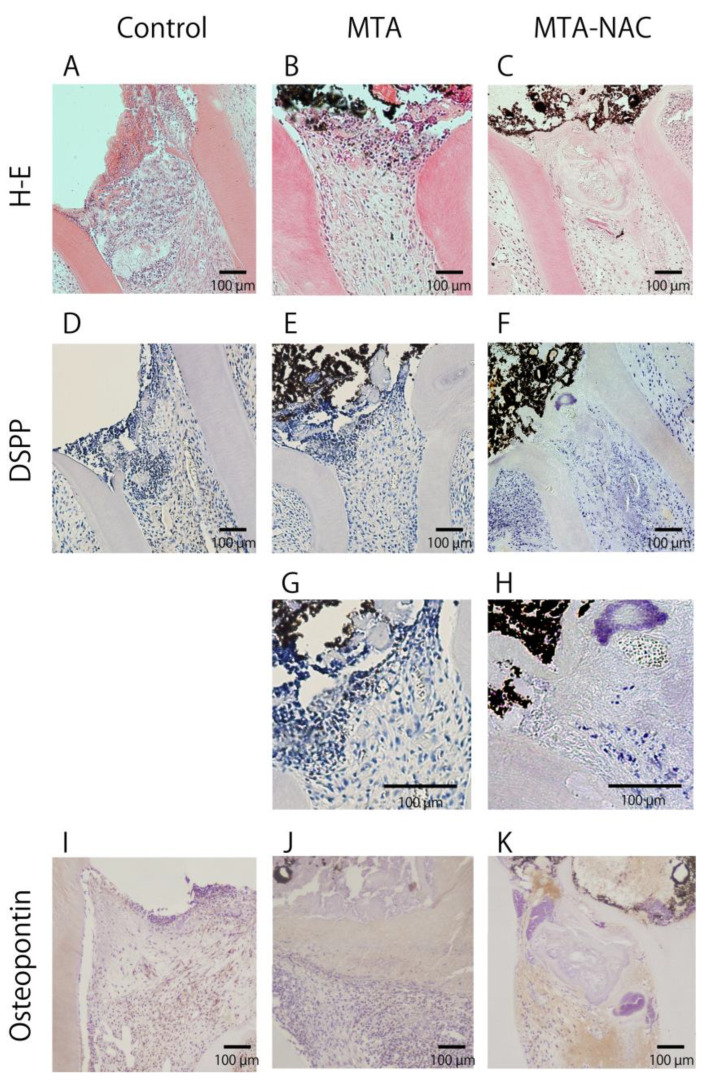
Histological evaluation of pulp 3 days postpulpotomy. (**A**–**C**): Hematoxylin and eosin-stained teeth. (**D**–**H**): DSPP-immunostained teeth. (**I**–**K**): Osteopontin-immunostained teeth.

In the control group, H–E staining revealed a heightened inflammatory response just below the pulp cut surface and throughout the upper part of the root canal (Figure 2A). The MTA group exhibited a moderate inflammatory response localized beneath the pulp cut surface (Figure 2B), whereas the MTA–NAC group showed only mild inflammatory cell infiltration in the same area (Figure 2C). Immunohistochemical staining for DSPP revealed no DSPP-positive areas in the control and MTA groups (Figure 2D,E). In contrast, the MTA–NAC group showed mildly DSPP-positive areas just below the pulp cut surface (Figure 2F). Figure 2G,H show the magnified details of Figure 2E,F. Immunohistochemical staining for osteopontin indicated no osteopontin-positive areas in the control group (Figure 2I). However, the MTA group displayed osteopontin-positive areas beneath the pulp cut surface (Figure 2J), whereas the MTA–NAC groups showed these areas below the pulp cut surface and above the root canal (Figure 2K).

#### 3.2.2. Pulp Changes at Seven Days Postpulpotomy

**Figure 3:**
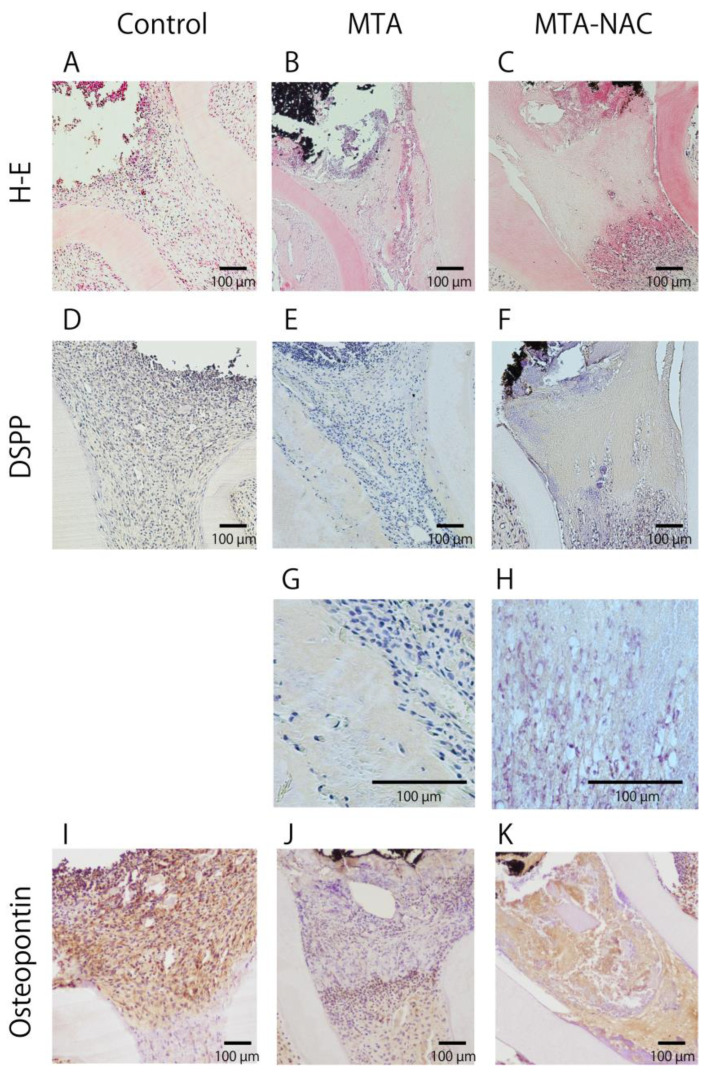
Histological evaluation of the pulp 7 days postpulpotomy. (**A**–**C**): Hematoxylin and eosin-stained teeth. (**D**–**H**): DSPP-immunostained teeth. (**I**–**K**): Osteopontin-immunostained teeth.

In the control group, H–E staining revealed a moderate inflammatory response below the pulp cut surface and above the root canal (Figure 3A). The MTA group showed a mild inflammatory response localized just below the pulp cut surface (Figure 3B), whereas the MTA–NAC group exhibited slight inflammatory cell infiltration below this surface alongside dentin bridge formation (Figure 3C). Immunohistochemical staining for DSPP revealed no DSPP-positive areas in the control group (Figure 3D). Conversely, the MTA group displayed DSPP-positive areas beneath the pulp cut surface and along the root canal wall (Figure 3E), whereas the MTA–NAC group exhibited extensive DSPP-positive areas in the formed dentin bridges (Figure 3F). Figure 3G,H show the magnified details of Figure 3E,F. Immunohistochemical staining for osteopontin highlighted osteopontin-positive areas just below the pulp cut surface in the control group (Figure 3I), beneath the pulp cut surface and above the root canal in the MTA group (Figure 3J), and below the pulp cut surface to the upper part of the root canal in the MTA–NAC group (Figure 3K).

## 4. Discussion

Pulpotomy is commonly employed in deciduous teeth and as an interim measure in immature permanent teeth. Due to the incomplete root development and delicate tooth structure of immature permanent teeth, definitive restorative procedures are often deferred in favor of temporary interventions like Atraumatic Indirect Pulp Capping, allowing for continued root maturation [19]. However, pulp treatment may be necessary in cases involving deep caries [4] or traumatic crown fractures [20]. Vital pulp therapy, which emphasizes the importance of the pulp for tooth longevity [21], is increasingly garnering attention. This therapy aims to preserve the pulp by indirectly or directly protecting it using pulp capping materials, with pulpotomy being one such method [22].

Pulpotomy involves substantial removal of the tooth structure and pulp amputation, often leading to pulpitis and less stable prognoses. To improve outcomes, controlling inflammation and promoting early dentin bridge formation are crucial [23]. Currently, calcium hydroxide preparations and MTA cement are the most common pulpotomy materials. Both materials initially form a necrotic layer on the cut surface, followed by inflammatory cell infiltration below this layer, granulation tissue formation, and eventually odontoblast-like cell arrangement, resulting in dentin bridge formation [24]. MTA cement generally offers a better prognosis than that provided by calcium hydroxide preparations owing to its superior sealing properties and slightly lower pH [25,26]. However, MTA exhibits strong alkalinity, with a pH of 12 when cured in water, leading to necrotic layer formation and aneutrophic calcification in cement-contacting areas. Thus, to improve prognoses, materials that minimize strong alkali irritation in the pulp while continuing to allow calcification through alkali effects are required [27,28].

To mitigate this issue, N-acetylcysteine (NAC), an antioxidant amino acid, was incorporated into MTA cement to lower its pH and reduce strong alkali stimulation. Although MTA cement powder alone dissolved in culture medium had a pH of 12.22, adding 250 mM NAC reduced the pH to 10.60. The H–E staining results on days 3 and 7 postpulpotomy revealed a less pronounced inflammatory response in the MTA–NAC group compared with the control and MTA groups, likely due to reduced strong alkali stimulation. Additionally, NAC is reported to exert anti-inflammatory effects by inhibiting the expression of TNF-α, an inflammatory cytokine [18], and reducing levels of prostaglandin E_2_, an inflammatory substance induced by resin components [29]. These findings suggest that NAC may offer anti-inflammatory benefits.

DSPP is the most abundant noncollagenous protein in dentin, synthesized and secreted by odontoblasts. The DSPP gene also serves as a marker for the differentiation of pulp-derived stem cells into odontoblasts [30,31]. In this study, the MTA–NAC group exhibited DSPP positivity on day 3, and by day 7, extensive DSPP positivity and dentin bridge formation were observed. The MTA group showed DSPP positivity on day 7, extending not only beneath the pulp cut surface but also along the root canal wall. Dentin sialoprotein, derived from DSPP, is involved in the early calcification of dentin, whereas dentin phosphoprotein, also processed from DSPP, plays a role in dentin maturation [32]. NAC may enhance dentin calcification and maturation by stimulating DSPP expression.

Dentin bridge formation is crucial for a successful outcome after pulpotomy. Reports indicate that dentin bridge formation with calcium hydroxide preparations can take 6 weeks to 6 months [33,34]. In this study, MTA–NAC application resulted in thick dentin bridges by day 7, which is a promising outcome for pulp capping materials and essential for preserving the root pulp. However, the present study did not assess long-term prognoses; hence, further long-term prognostic studies are required.

Osteopontin, a noncollagenous protein deposited at the boundary between dentin and repair dentin [35], is involved in the secretion of type I collagen, a major organic component of dentin [36]. In the present study, the MTA–NAC group displayed extensive osteopontin-positive areas, suggesting that NAC may induce type I collagen formation and promote calcification through osteopontin activation.

Bacterial infection in the pulp is a major factor affecting prognosis after pulpotomy [37]. The noninfectious model used in this study does not account for bacterial infection. In vitro studies have shown that MTA does not inhibit pulp cell proliferation when cocultured with human pulp cells and exposed to bacteria, whereas calcium hydroxide triggers such inhibition [38]. Given its antimicrobial properties [16], NAC may offer a better prognosis than MTA, even in the presence of bacterial infection.

To investigate the potential benefits of an antioxidant amino acid on MTA, which is known for its exceptional prognosis among current pulp capping options, NAC was incorporated into MTA to perform pulpotomy in rats. The hardening and swelling of MTA contribute to its superior sealing properties, while the release of calcium and hydroxide ions induces hard tissue formation and provides antibacterial effects. These properties are well documented. However, MTA also induces necrotic layer formation and strong inflammatory cell infiltration at the pulp contact surface. Adding NAC to MTA reduced pulp inflammation by lowering the pH, which did not inhibit hard tissue formation, but rather, enhanced it. This improvement makes MTA–NAC a more desirable pulp capping material. Its strong calcification-inducing ability may be applicable not only for pulpotomy but also for staged caries removal and selective (incomplete) dentin removal, reflecting current global trends. Future research should focus on its use as an indirect covering material and the permeability of dentin tubules.

Furthermore, the clinical translation of MTA-NAC necessitates a deeper understanding of its interaction with the dentin–pulp complex under various physiological and pathological conditions. While this study demonstrated promising outcomes in a non-infected model, the dynamic interplay between the material and the host’s immune response in the presence of bacterial challenges warrants further investigation. Specifically, the evaluation of cytokine release profiles, macrophage polarization, and the activation of Toll-like receptors (TLRs) would provide valuable insights into the immunomodulatory effects of MTA-NAC.

### 4.1. Limitations

This study has several limitations that should be acknowledged. Firstly, we did not evaluate the physicochemical properties of the experimental material, MTA-NAC. While favorable long-term prognoses in clinical applications are linked to robust physicochemical characteristics, this study did not assess properties such as compressive strength, setting time, and solubility, which are essential for determining the material’s suitability for clinical implementation and ensuring its long-term viability and performance. Secondly, although this study demonstrated promising outcomes in a non-infected model, it did not account for the influence of bacterial infection, a major factor affecting prognosis after pulpotomy. Further assessment and enhancement of physical properties and antimicrobial activity are needed to advance NAC’s clinical application. Finally, we did not assess the long-term prognosis of pulpotomy using MTA-NAC; therefore, further long-term prognostic studies are required.

### 4.2. Prospects of Further Research

The clinical translation of MTA-NAC necessitates a deeper understanding of its interaction with the dentin–pulp complex under various physiological and pathological conditions. While this study demonstrated promising outcomes in a non-infected model, the dynamic interplay between the material and the host’s immune response in the presence of bacterial challenges warrants further investigation. Specifically, the evaluation of cytokine release profiles, macrophage polarization, and the activation of Toll-like receptors (TLRs) would provide valuable insights into the immunomodulatory effects of MTA-NAC. Moreover, the long-term stability and durability of the dentin bridge formed by MTA-NAC should be assessed using advanced imaging techniques such as micro-CT and confocal microscopy. These methods would enable a detailed analysis of the mineral density, structural integrity, and permeability of the newly formed dentin. Additionally, the examination of the interface between the material and the dentin, focusing on the formation of a hybrid layer and the presence of microleakage, is crucial for predicting clinical success. In addition to the biological aspects, the handling characteristics and ease of use of MTA-NAC in a clinical setting should be considered. Factors such as setting time, viscosity, and adaptability to different cavity configurations are essential for practical application. Future studies should aim to optimize the formulation of MTA-NAC to enhance its clinical performance and ensure consistent and predictable outcomes in diverse clinical scenarios. Finally, cost-effectiveness should be considered for the clinical application of MTA-NAC. Comparative studies with other pulp capping materials, including a cost–benefit analysis, are needed to see if MTA-NAC is a better choice for clinical use.

## 5. Conclusions

This study demonstrated that MTA-NAC reduced pulp inflammation and promoted superior dentin bridge formation compared to MTA. At 3 days, MTA-NAC showed milder inflammation and earlier signs of dentin bridge formation. By day 7, MTA-NAC exhibited thick dentin bridges and extensive DSPP positivity. MTA-NAC appears to be a promising pulp capping material for pulpotomy.

## Figures and Tables

**Table 1:** Materials used in this study.

Materials	Composition	Manufacturer
Mielle (MTA cement)	Calcium silicate, calcium aluminate, zirconium oxide, silicon dioxide	YAMAKIN, Kochi, Japan
Superbond C&B	Powder: PMMA Liquid: MMA, 4-META	Sun Medical

**Table 2:** Groups of pulp capping material.

Group	Pulp Capping Material
Control	Superbond C&B
MTA	Mielle (0.12 g of powder and 0.03 g of purified water)
MTA-NAC	Mielle (0.12 g of powder and 0.03 g of purified water) mixed with NAC (50 mg)

## Data Availability

The original contributions presented in the study are included in the article, further inquiries can be directed to the corresponding author.
